# Place of death, care-seeking and care pathway progression in the final illnesses of children under five years of age in sub-Saharan Africa: a systematic review

**DOI:** 10.7189/jogh.09.020422

**Published:** 2019-12

**Authors:** Jessica Price, Joseph Lee, Merlin Willcox, Anthony Harnden

**Affiliations:** 1 Nuffield Department of Primary Care Health Sciences, University of Oxford, Oxford, UK; 2 Department of Primary Care and Population Medicine, University of Southampton, Southampton, UK

## Abstract

**Background:**

Half of all under-5 deaths occur in sub-Saharan Africa. Reducing child mortality requires understanding of the modifiable factors that contribute to death. Social autopsies collect information about place of death, care-seeking and care-provision, but this has not been pooled to learn wider lessons. We therefore undertook a systematic review to collect, evaluate, map, and pool all the available evidence for sub-Saharan Africa.

**Methods:**

We searched PubMed, Embase, Global Health, the Cochrane Library and grey literature for studies relating to under-5 deaths in sub-Saharan Africa with information on place of death and/or care-seeking during a child’s final illness. We assessed study quality with a modified Axis tool. We pooled proportions using random effects meta-analysis for place of death and for each stage of the Pathways to Survival framework. Pre-specified subgroup analysis included age group, national income and user-fee policy. We explored heterogeneity with meta-regression. Our protocol was published prospectively (CRD42018111484).

**Results:**

We included 34 studies from 17 countries. Approximately half of the children died at home, irrespective of age. More children died at home in settings with user-fees (69.1%, 95% confidence interval (CI) = 56.2-80.6, I^2^ = 98.4%) compared to settings without user-fees (43.8%, 95% CI = 34.3-53.5, I^2^ = 96.7%). Signs of illness were present in over 95% of children but care-seeking differed by age. 40.1% of neonates (95% CI = 20.7-61.3, I^2^ = 98.0%) died without receiving any care, compared to 6.4% of older children (95% CI = 4.2%-9.0%, I^2^ = 90.6%). Care-seeking outside the home was less common in neonatal deaths (50.5%, 95% CI = 35.6-65.3, I^2^ = 98.3%) compared to infants and young children (82.4%, 95% CI = 79.4%-85.2%, I^2^ = 87.5%). In both age groups, most children were taken for formal care. Healthcare facilities discharged 69.6% of infants and young children who arrived alive (95% CI = 59.6-78.7, I^2^ = 95.5%), of whom only 34.9% were referred for further care (95% CI = 15.1-57.9, I^2^ = 98.7%).

**Conclusions:**

Despite similar distributions in place of death for neonates and infants and young children, care-seeking behaviour differed by age groups. Poor illness recognition is implicated in neonatal deaths, but death despite care-seeking implies inadequate quality care and referral for older children. Understanding such care-seeking patterns enables targeted interventions to reduce under-5 mortality across the region.

The under-5 mortality rate in sub-Saharan Africa continues to be the highest in the world, with half of all under-5 deaths in 2017 occurring in this region despite representing only 13.9% of the global population [1,2]. The United Nations used Sustainable Development Goal 3.2 to set a target under-5 mortality rate of 25 deaths per 1000 live births by 2030 [3]. However, weak vital registration systems in many countries across sub-Saharan Africa make it difficult to accurately estimate the number of births and deaths in a given year. This difficulty is compounded when births and deaths occur outside the health care system. Population based studies have shown that high proportions of under-5 deaths occur outside health facilities [4-6] though no work has yet attempted to systematically identify and pool these findings across countries in sub-Saharan Africa.

Place of death is just one social circumstance of death which can offer insights into modifiable factors contributing to under-5 mortality. There has been increasing recognition of the importance of understanding the care-seeking behaviours and barriers faced by caregivers during a child’s final illness. Traditionally these were explored by facility-based audits or confidential enquiries, but the past decade has seen a rise in the use of social autopsies as a population-based tool to investigate social circumstances of death [7]. A social autopsy is a structured interview with the caregiver of the deceased which attempts to make a “social diagnosis” of cause of death by identifying its cultural, social and health-systems antecedents [5,8,9]. Different social autopsy tools and frameworks for analysis have been developed [7].

The Pathways to Survival Framework is a commonly-used analytic framework in the study of care pathways in fatal childhood illness. First developed in 1996 and subsequently adapted, it provides a means of thinking through the processes of seeking and providing care both within and outside the home during a child’s illness [10]. Despite the growing number of studies using this framework, individual studies do not give a sense of whether patterns identified apply across sub-Saharan Africa, nor whether there are characteristics which influence care-seeking processes universally.

In this review we aim to systematically gather and appraise all available evidence to 1) determine the proportion of under-5 deaths occurring within and outside health facilities in sub-Saharan Africa, 2) characterize the processes of seeking and providing care during the fatal illness, as defined by the Pathways to Survival framework and 3) to determine whether place of death or care-seeking behavior varied based on the age of the child, income status of the country or presence of user-fees.

## METHODS

### Search strategy and selection criteria

The systematic review protocol was prospectively published and is available from PROSPERO (PROSPERO 2018 CRD42018111484, http://www.crd.york.ac.uk/PROSPERO/display_record.php?ID=CRD42018111484).

We conducted a systematic search of the literature to identify studies relating to deaths of children under 5 years of age from sub-Saharan Africa, that included information on place of death and/or care-seeking behaviour during a child’s final illness. We searched PubMed, Embase, Global Health and the Cochrane Library using the following search terms in various combinations: care pathway*, pathways to care, care-seeking, health care-seeking, treatment seeking, “pathway to survival”, “three delay”, social autopsy, verbal autopsy with terms to identify children under years (child, under-5 under 5, infant, neonat*, newborn) and countries in sub-Saharan Africa as defined by the World Bank [11]. Mesh terms were included where appropriate. The final search was conducted on 15 August 2018. We also conducted a manual review of the reference lists of relevant articles to identify any additional resources that should be included and contacted leading authors in the field to identify relevant unpublished data.

We included studies if they were conducted in a country in sub-Saharan Africa, reporting on deaths in children under-5 years, and included data on at least one of the following:

1. The number or proportion of children who died at home
2. The number or proportion of children who died in a health facility
3. Treatment seeking behavior during the final illness, including one or more of the stages of care as identified by the pathways to survival framework (ie, recognition of illness, provision of care in the home, care-seeking outside the home from formal and informal providers, referral and acceptance of referral).

We excluded studies that focused only on stillbirths or did not separate stillbirths from neonatal or under-5 deaths, or if results were reported purely qualitatively. Review articles were not included as this systematic review focused on primary research, however they were used to confirm that relevant references were identified and included in the analysis. We didn’t exclude articles based on publication date or language.

Two reviewers (JP and JL) independently screened all titles and abstracts for relevance, then independently selected full texts for inclusion in the final review. Discrepancies were resolved by consensus. Data from each included study were independently extracted by the same reviewers and cross checked using a standardized template to record details including country; age group (neonatal/1-59 months/under-5; total number of deaths; number of deaths at home, in health facilities, on route to facilities or elsewhere; and care-seeking data (broken down into the stages identified by the Pathways to Survival framework). Where the published manuscript did not disaggregate the data in a way that could be aligned to the criteria we used in this review, authors were contacted to ask for a more detailed breakdown of the original data.

### Quality appraisal

We adapted the AXIS tool for cross-sectional studies to allow us to assess study quality [12] (Appendix 1 in **Online Supplementary Document**). This was necessary as we included studies with different objectives to our own as long as they reported data we could use. We adapted the scoring system retaining points relevant to data collection but removing points not relevant to our objectives (and thus to assessing those aspects of the study relevant to the inclusion of the data in the meta-analysis) eg, were the author’s discussions and conclusions justified by the results. Quality indicators were also re-worded from the negative to the affirmative such that the presence of each indicator (which demonstrated improved quality) was associated with a higher score. For studies that had different aims and objectives to ours, we evaluated each quality indicator in relation to the aims and objectives of this review rather than the aim of the original study. Two reviewers independently assessed each study, awarding 1 point where the criterion was met, and resulting in a final score calculation as a proportion between 0 (poor quality) and 1 (high quality). Conflicts were agreed with discussion.

### Statistical analysis

We pooled proportions using random effects meta-analysis, after the Freeman-Tukey Double Arcsine Transformation of variances, using Stata command *metaprop* [13]. We used score (Wilson) estimation to calculate 95% confidence intervals. We used the I^2^ statistic as our measure of statistical heterogeneity. We estimated proportions in pre-specified sub-groups according to age group (neonatal or infants and young children), national income status (low income, lower middle income and upper middle income), and the presence of user-fees in each country (yes/no). Where studies only reported results for under-5 deaths overall, and we were unable to get an appropriate age breakdown after contacting the authors of the original publication, we grouped this data with results for infants and young children.

We used random-effects meta-regression to explore heterogeneity, assessing the association between each outcome and age group, national income status and user-fee policy, using the Stata command *metareg* [14].

## RESULTS

### Search

**Figure 1:**
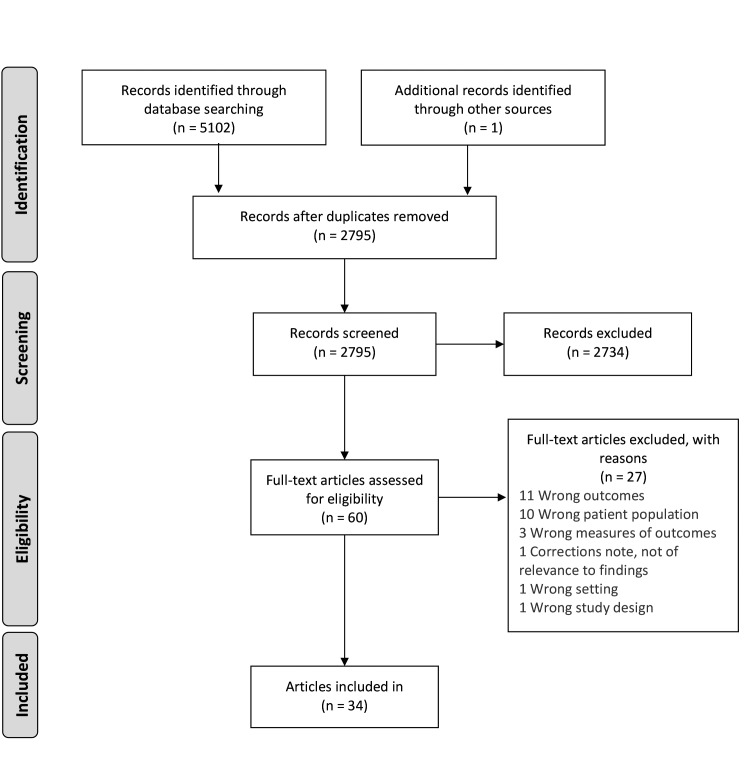
PRISMA flow diagram [15].

The literature search found 5102 citations. After deduplication, 2794 citations remained for title and abstract screening. In addition, our review of reference lists identified one further research report that was included giving a total 2795 abstracts. After two reviewers independently screened all titles and abstracts, 60 studies were included for full text review, and 34 were included in the final analysis. (**Figure 1**).

### Included studies characteristics

**Figure 2:**
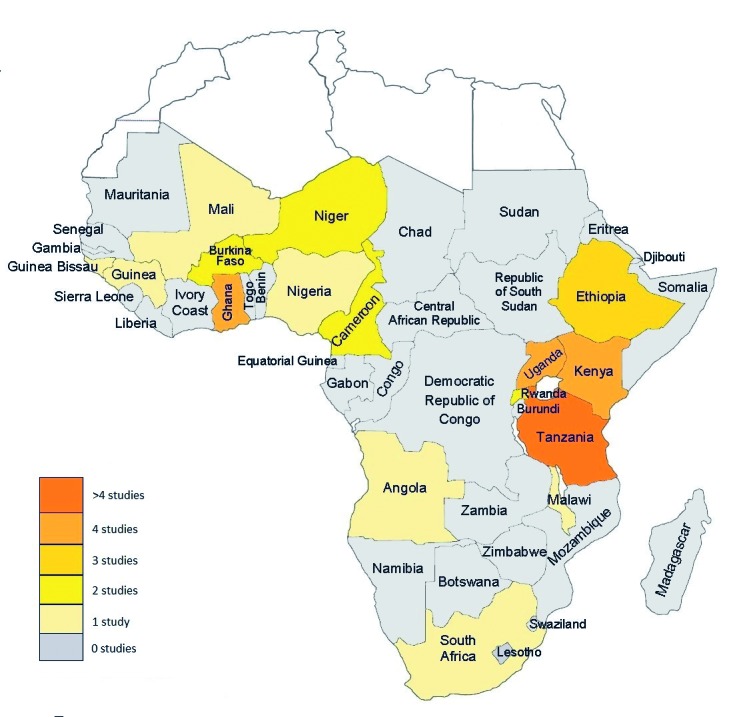
Map showing number and location of included studies.

Characteristics of the included studies are summarised in **Table 1**. Sixteen studies reported on neonatal deaths, 22 on deaths of infants and young children and seven on overall under-5 deaths. Data was drawn from 17 countries (**Figure 2**), 13 low-income countries, 3 lower middle income countries and 1 upper middle income country. Sixteen studies were conducted in settings where user-fees are charged at government facilities at the point of care, while 21 were from settings where governments had abolished user-fees. In one study in Ghana [31], the user-fee policy was changed during the observation period, and in Nigeria [4] user-fee policy varies across states [47], and so both studies were excluded from the relevant sub-analysis.

**Table 1:** Characteristics of included studies

First author and year of publication	Period of data collection	Country	Age	National income status	User-fees (Yes/ No/ Mixed)	Setting (Rural/ Urban/ Mixed)	Total deaths	Instrument used	Recall period	Data collected on the care-seeking process 1) illness recognition; 2) recognition of severe illness; 3) home care; 4a) sought care outside the home; 4b) type of health care sought; 5) referral; 6) compliance referral advice; 7) place of death	Conceptual model used to drive analysis	Quality score
Snow 1994 [16]	1992-1993	Kenya	1-59 mo	Low income	Y	Rural	137	Site-specific pro-forma	Unspecified	7	None	0.71
Sodemann 1997 [17]	1992-1993	Guinea-Bissau	1-30 mo	Low income	Y	Urban	125	Unspecified	Median 7 mo (IQR 6-9 mo)	4a, 4b, 7	None	0.50
Garg 2001 [18]	1998	Kenya	0-59 mo	Low income	Y	Rural	97	Unspecified	Median 29 d (range 7-152 d)	4b, 7	None	0.57
Armstrong Schellenberg 2002 [19]	1997-2000	Tanzania	1-59 mo	Low income	N	Rural	427	Unspecified	6 weeks-3 mo	4a, 4b, 7	None	0.71
Schumacher 2002 [20]	1998-1999	Guinea	0-59 mo	Low income	Y	Rural	133	BASICS/JHU standardised verbal autopsy and social autopsy questionnaires	Unspecified	1, 2, 3, 4a, 4b, 7	Pathways to Survival	0.93
Hinderaker 2003 [21]	1995-1996	Tanzania	0-27 d	Low income	N	Rural	76	Medical interviews plus study-specific VA questionnaire	Unspecified	4a, 7	None	0.57
Muller 2003 [22]*	1999	Burkina Faso	11-32 mo	Low income	Y	Rural	17	Physician interview, non standardised	6 mo	3, 4a, 4b	None	0.64
de Savigny 2004 [23] *	1999-2001	Tanzania	0-59 mo	Low income	N	Rural	320	National Sentinel System VA tool	2 weeks	3, 4a	None	0.93
Beiersmann 2007 [24]*	1999-2002	Burkina Faso	0-59 mo	Low income	Y	Rural	100	Unspecified	1-3 mo	4b	None	0.29
Kamugisa 2007 [25]	Unspecified	Tanzania	0-59 mo	Low income	N	Rural	53	WHO VA 1999	2 y	7	None	0.79
Edmond 2008 [26]	2003-2004	Ghana	0-28 d	Low income	Y	Rural	590	WHO VA 1999	6 mo	4a, 4b, 7	None	0.71
Hildenwall 2008 [27] *	2006	Uganda	1-59 mo	Low income	N	Rural	26	INDEPTH Network VA merged with the social autopsy from the Bolivian Mortality Survey	4-6 weeks	4a, 4b, 7	Pathways to Survival	0.79
Kallander 2008 [28] *	2005-2007	Uganda	1-59 mo	Low income	N	Rural	44	INDEPTH Network VA merged with the social autopsy from the Bolivian Mortality Survey	4-6 weeks	2, 3, 4a, 4b, 5, 6, 7	None	0.86
Kaatano 2009 [29] *	2006	Tanzania	1-59 mo	Low income	N	Rural	141	WHO VA 2007	10 y	1, 3, 4a, 4b, 7	None	0.50
Waiswa 2010 [30]	2005-2008	Uganda	0-27 d	Low income	N	Rural	64	VASA questionnaire blends INDEPTH Network VA merged with CHERG SA questionnaire	4-6 weeks	4a, 4b, 7	Three Delays Model	0.86
Kallander 2011 [5]	2008-2010	Ghana Uganda	1-59 mo	Ghana: low income Uganda: low income	Ghana: N Uganda: N	Ghana: rural Uganda: rural	Ghana: 40 Uganda: 434	INDEPTH Network Social Autopsy for child deaths	6 mo	1, 2, 3, 4a, 4b, 5, 6, 7	Pathways to Survival & Three Delays Model	0.71
Manortey 2011 [31]	2005-2010	Ghana	0-27 d, 1-59 mo	Low income	Mixed	Rural	118	WHO VA 2007	5 y	7	None	0.86
Mrisho 2012 [32]	2007	Tanzania	0-27 d	Low income	N	Rural	219	INDEPTH Network VA	3 y	4a, 7	None	0.71
Olack 2014 [33]*	2007-2010	Kenya	0-59 mo	Low income	Y	Urban	309	Site specific - interview conducted with household proxy after death	Unspecified	4a	None	0.57
Rahman 2014 [34] *	2000-2012	Ghana Ethiopia Tanzania Uganda	1-59 mo	Ghana: low income Ethiopia: low income Tanzania: low income Uganda: low income	Ghana: N Ethiopia: Y Tanzania: N Uganda: N	Ghana: rural Ethiopia: mixed Tanzania: rural Uganda: rural	Ghana: 145 Ethiopia: 61 Tanzania: 38 Uganda: 103	WHO verbal autopsy tool - versions varied by site	Unspecified	4a	None	0.21
Koffi 2015 [35]	2012	Cameroon	0-27 d	Lower middle income	Y	Mixed	164	VASA questionnaire blends the Population Health Metrics Research Consortium (PHMRC) VA questionnaire with the CHERG SA questionnaire	4 y	1, 2, 3, 4a, 4b, 5, 6, 7	Pathways to Survival	0.86
Koffi 2015 [36]	2013	Malawi	0-27 d	Low income	N	Mixed	380	VASA questionnaire blends the Population Health Metrics Research Consortium (PHMRC) VA questionnaire with the CHERG SA questionnaire	4 y	1, 2, 3, 4a, 4b, 5, 6, 7	Pathways to Survival	0.79
Assefa 2016 [37]	2008-2013	Ethiopia	0-27 d	Low income	Y	Mixed	301	WHO VA 2007	1-3 mo	7	None	0.79
D'Ambruoso 2016 [38]	2012-2013	South Africa	0-27 d, 1-59 mo	Upper middle income	N	Rural	110	WHO VA 2012	Unspecified	1, 4b	Thematic coding framework home-to-hospital pathway	0.79
Kalter 2016 [39]	2007-2010	Niger	0-27 d	Low income	N	Mixed	453	VASA questionnaire blends the Population Health Metrics Research Consortium (PHMRC) VA questionnaire with the CHERG SA questionnaire	4 y	1, 2, 3, 4a, 4b, 5, 6	Pathways to Survival	0.86
Koffi 2016 [6]	2012	Niger	1-59 mo	Low income	N	Mixed	620	VASA questionnaire blends the Population Health Metrics Research Consortium (PHMRC) VA questionnaire with the CHERG SA questionnaire	Mean 2.7 y (range 2-5 y)	1, 2, 3, 4a, 4b, 5, 6, 7	Pathways to Survival	1.00
Obor 2016 [40]	2003-2012	Kenya	0-28 d	Low income	Y	Rural	533	Unspecified	Unspecified	4a, 4b	None	0.43
Rosario 2016 [41]	2009-2010	Angola	0-27 d, 1-59 mo	Lower middle income	N	Mixed	340	INDEPTH/ WHO VA 1999	6 weeks	7	None	0.79
Bogale 2017 [42]	2016	Ethiopia	0-28 d	Low income	Y	Mixed	37	INDEPTH Network VA and SA	18 mo	4a	Three Delays Model	0.86
Koffi 2017 [4]	2009-2013	Nigeria	1-59 mo	Lower middle income	Mixed	Mixed	2057	VASA questionnaire blends the Population Health Metrics Research Consortium (PHMRC) VA questionnaire with the CHERG SA questionnaire	5 y	1, 2, 3, 4a, 4b, 5, 6, 7	Pathways to Survival	1.00
Koffi 2017 [43]	2012	Cameroon	1-59 mo	Lower middle income	Y	Mixed	635	VASA questionnaire blends the Population Health Metrics Research Consortium (PHMRC) VA questionnaire with the CHERG SA questionnaire	5 y	1, 2, 3, 4a, 4b, 5, 6, 7	Pathways to Survival	0.93
Navale 2017 [44]	2013-2014	Rwanda	0-11 mo	Low income	Y	Rural	133	VASA tool merging WHO VA 2012, Rwanda MOH's Death Audit Tool and the 2010 Rwanda Demographic and Health Survey	Unspecified	1, 2, 4a, 4b, 7	Unique to this study - created a driver framework for infant mortality from review of the literature.	0.57
Kagabo 2018 [45]	2013-2014	Rwanda	0-27 d, 1-59 mo	Low income	Y	Rural	618	VASA tool merging WHO VA 2012, Rwanda MOH's Death Audit Tool and the 2010 Rwanda Demographic and Health Survey	Unspecified	1, 4a, 4b, 7	Behavioural model of predictors of care-seeking: predisposing factors, enabling resources, need, care-seeking behaviour	0.93
Willcox 2018 [46]	2011-2015	Mali Uganda	0-27 d, 1-59 mo	Mali: low income Uganda: low income	Mali: Y Uganda: N	Mali: mixed Uganda: mixed	Mali: 762 Uganda: 313	Semi-structured qualitative interview plus modified QUARITE questionnaire	Unspecified	1, 2, 3, 4a, 4b, 5, 6, 7	None	0.64

CI – confidence interval, d – day, mo – months, y – years

*These studies report on mortality related to diarrhoeal disease, pneumonia, acute febrile illnesses, or malaria only. All other studies report all-cause mortality.

### Quality appraisal

Study quality ranged from 0.21-1.00 (Table S1 in **Online Supplementary Document**). The most common problems identified in the included studies were failing to report on all stages of the care pathway, failing to report the response rate and failing to categorise non-responders where the response rate was less than 90%. Appropriate categorisation of non-responders included descriptions of age and other demographic details, and reasons for non-participation to allow an assessment of risk of bias.

### Synthesis of results

Proportions varied between studies, and statistical heterogeneity in pooled estimates as estimated by I^2^ was high (Table S2 in **Online Supplementary Document**).

### Place of death

**Figure 3:**
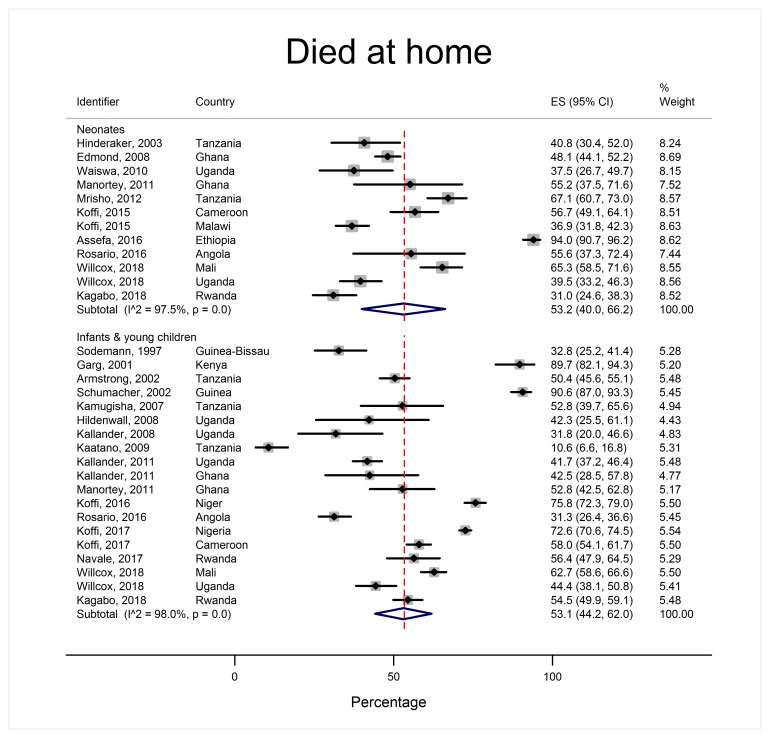
Proportion of children who died at home, by age group.

As shown in **Table 2** and **Figure 3**, place of death was similar across age groups. Approximately half of all children died at home (53.2% of neonates 95% confidence interval (CI) = 40.0-66.2, I^2^ = 97.5% and 53.1% of infants and young children 95% CI = 44.2-62.0, I^2^ = 98.0% respectively) and over a third died in health facilities (37.8% of neonates, 95% CI = 26.4%-49.8%, I^2^ = 97.0% and 33.4% of infants and young children, 95% CI = 25.5%-41.9%, I^2^ = 97.6%). Small fractions of deaths happened *en route* to a health care facility or elsewhere.

**Table 2:** Summary of pooled results, stratified by age group

Outcome	Neonates					Infants and young children				
	Number of studies*	**Number of deaths**	**Proportion**	**95% CI**	**I^2^**	Number of studies*	**Number of deaths**	**Proportion**	**95% CI**	**I^2^**
Died at home	12	2373	53.2	40.0-66.2	97.5	17	6803	53.1	44.2-62.0	98.0
Died in a health facility	12	2373	37.8	26.4-49.8	97.0	14	6153	33.4	25.5-41.9	97.6
Died *en route* to a health facility	4	577	5.1	3.3-7.1	0.0	7	3997	5.6	3.2-8.6	89.0
Died elsewhere	8	1444	3.4	0.6-7.7	89.5	11	5314	6.2	3.0-10.4	96.3
Signs/Symptoms of illness	7	1294	95.0	84.9-99.9	97.2	11	5345	98.2	95.2-99.9	97.0
Signs/Symptoms of severe illness	5	1097	84.3	60.0-98.5	98.8	9	5003	88.1	78.4-95.3	98.7
Died immediately/no care given†	6	1134	40.1	20.7-61.3	98.0	14	5692	6.4	4.2-9.0	90.6
Home care given†	5	1097	14.4	8.8-21.0	87.9	12	5392	52.6	37.8-67.1	99.0
Sought/attempted to seek care outside the home†	11	2695	50.5	35.6-65.3	98.3	22	7044	82.4	79.4-85.2	87.5
Sought/attempted to seek formal care†	12	2539	41.2	29.3-53.5	97.2	19	6500	73.1	66.5-79.2	96.5
Sought/attempted to seek informal care	9	1948	12.7	6.5-20.3	94.7	12	5565	23.5	14.2-34.3	98.6
Died before setting out/*en route* to provider	5	1097	5.8	3.5-8.6	66.4	8	4223	10.4	6.1-15.7	94.3
Arrived alive at formal health facility (out of all deaths)†	5	1097	36.5	25.2-48.5	93.8	9	4553	58.7	48.0-68.9	97.5
Arrived alive at formal health facility (of those who sought formal care)	5	455	85.8	76.5-93.1	84.0	9	2956	88.4	79.4-95.3	97.0
Left formal health facility alive (out of all deaths)†	5	1097	21.4	14.4-29.4	89.0	6	4180	42.7	30.4-55.4	98.3
Left formal health facility alive (of those who arrived alive)	5	385	62.9	40.9-82.4	94.6	6	2266	69.6	59.6-78.7	95.5
Referred for further care (out of all deaths)	5	1097	8.7	2.4-18.4	95.4	9	4681	17.9	9.5-28.2	98.3
Referred for further care (of those who left the facility alive)	4	29	30.7	7.1-61.3	94.7	5	136	34.9	15.1-57.9	98.7
Accepted referral (of all deaths)	5	1097	5.7	0.9-13.9	95.3	8	4584	11.9	6.7-18.3	96.6
Accepted referral (of those referred)	5	101	65.9	49.3-81.0	50.3	8	560	69.3	57.7-79.9	85.6

CI – confidence interval

*In cases where data was collected from more than one country as part of the same study, each country was counted as a separate study for the purpose of the meta-analysis

†Significant at *P* < 0.05.

The place of death did not vary significantly by national income status (Table S2 and Appendix S4 in **Online Supplementary Document**). Countries where user-fees were charged appeared to have a bimodal distribution of home deaths (Appendix S4 in **Online Supplementary Document**). Therefore, while a comparison of pooled proportions across settings with and without user-fees would suggest that user-fees are associated with a higher proportion of home deaths (Table S3 and Appendix S4 in **Online Supplementary Document**), this should be interpreted with caution.

### Care pathways analysis – curative care-seeking

**Figure 4:**
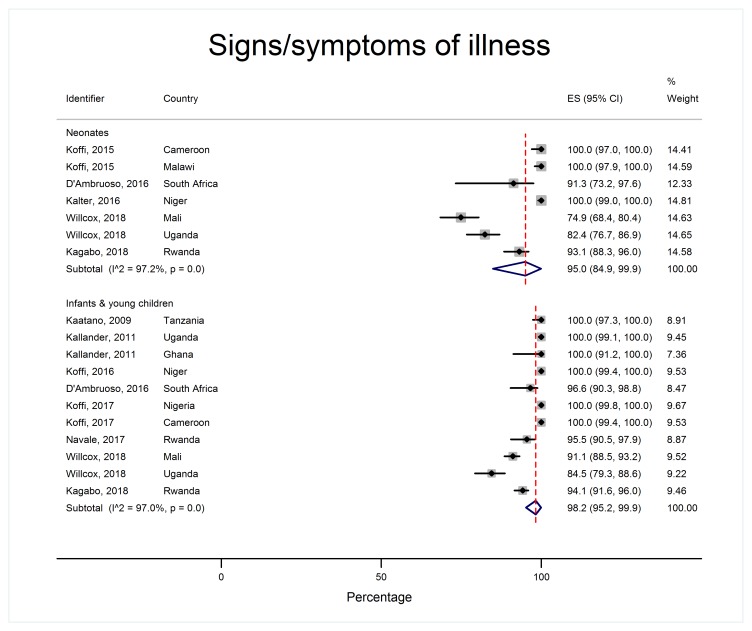
Proportion of children with signs/symptoms of illness, by age group.

**Figure 5:**
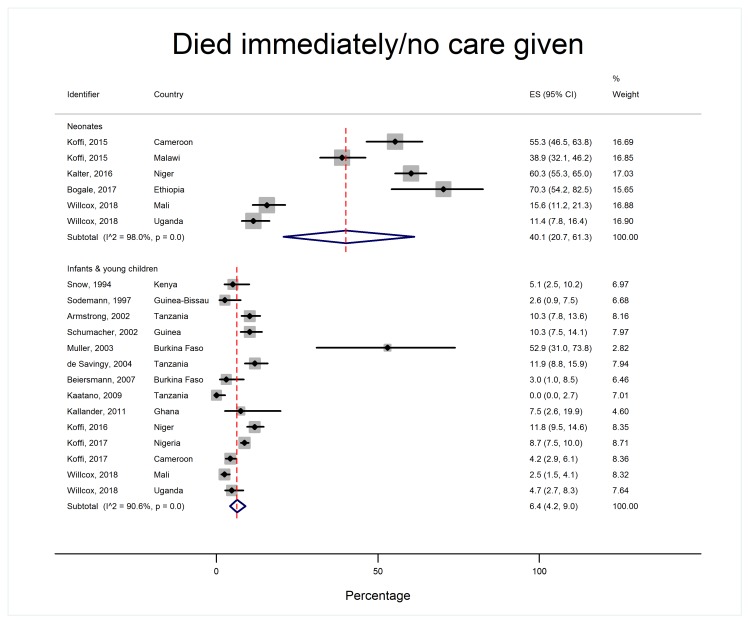
Proportion of children who died immediately or without receiving any care, by age group.

**Figure 6:**
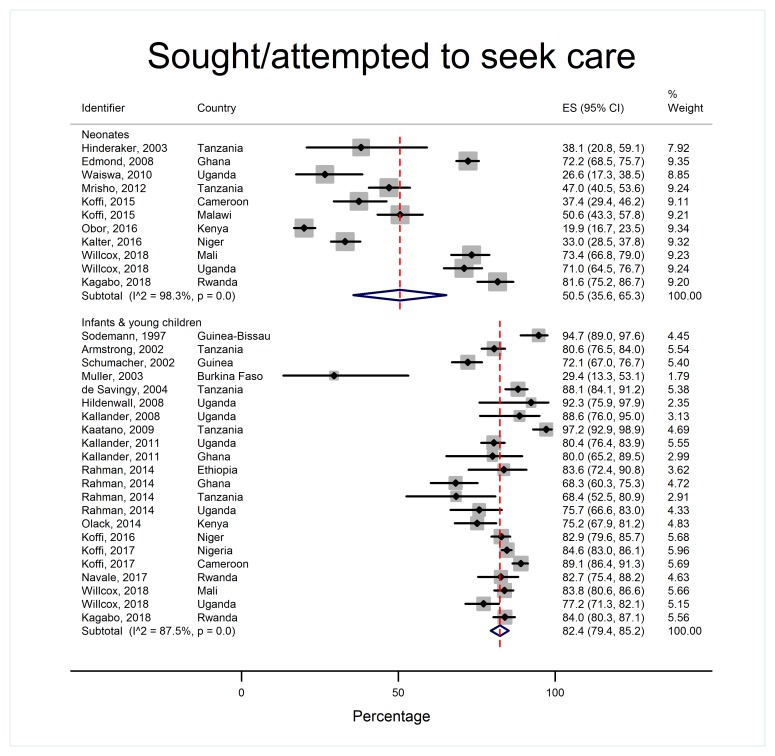
Proportion of caregivers who sought or attempted to seek care outside the home for their child during the final illness, by age group.

**Figure 7:**
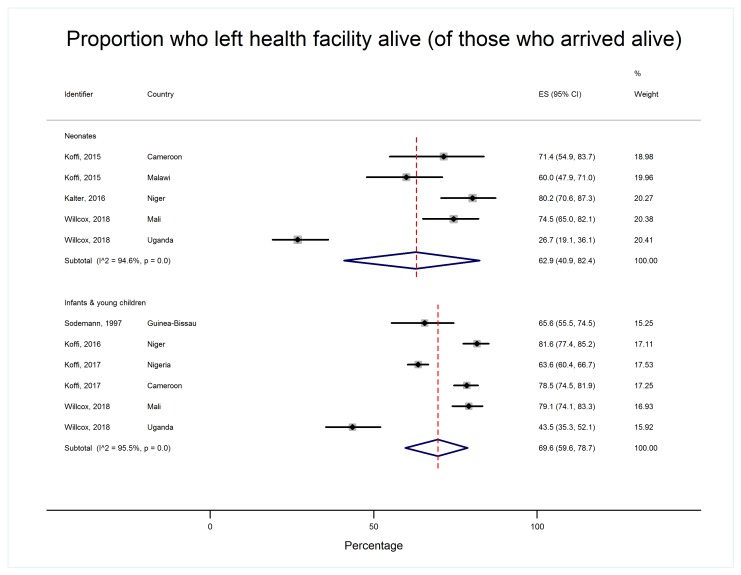
Proportion of children who left the first health facility alive, by age group.

Patterns of care-seeking were different for neonates and older children, as shown in **Table 2** and **Figures 4 to 7**.

#### Home and travel to care

Over 95% of caregivers of neonates and infants and young children recognized their child was unwell and over 80% reported potentially serious signs (**Figure 4**). However, 40.1% of neonates (95% CI = 20.7-61.3, I^2^ = 98.0%) died without care of any sort being provided, whereas very few older children died with no care at all (6.4% 95% CI = 4.2%-9.0%, I^2^ = 90.6%) (**Figure 5**). 14.4% of neonates (95% CI = 8.8%-21.0% I^2^ = 87.9%) received care in the home compared to over half of infants and young children (52.6%, 95% CI = 37.8%-67.1%, I^2^ = 99.0%). Care-seeking outside the home was less common in neonatal deaths (50.5%, 95% CI = 35.6-65.3, I^2^ = 98.3%) compared to infants and young children (82.4%, 95% CI = 79.4%-85.2%, I^2^ = 87.5%) (**Figure 6**). In both age groups, more children were taken for formal care (41.2% of neonates, 95% CI = 29.3%-53.5%, I^2^ = 97.2% and 73.1% of infants and young children, 95% CI = 66.5%-79.2% I^2^ = 96.5%) than informal care (12.7% of neonates, 95% CI = 6.5%-20.3%, I^2^ = 94.7%, and 23.5% of infants and young children, 95% CI = 14.2%-34.3%, I^2^ = 98.6%). A small minority of children died before setting out or arriving at a care facility (5.8% of neonates, 95% CI = 3.5%-8.6%, I^2^ = 66.4% and 10.4% of infants and young children, 95% CI = 6.1%-15.7%, I^2^ = 94.3%).

#### At care facilities

A minority of neonates in their final illnesses were alive when they arrived at a care facility (36.5% 95% CI = 25.2%-48.5%, I^2^ = 93.8%) whereas the majority of infants and young children made it to a formal health care facility alive (58.7%, 48.0%-68.9%, I^2^ = 97.5%). However, in both age groups, this represented a large majority of those who sought formal care (85.8% of neonates, 95% CI = 76.5%-93.1%, I^2^ = 84.0% and 88.4% of infants and young children, 95% CI = 79.4-95.3, I^2^ = 97.0%). Also, in both age groups most of those children who arrived alive left the care facility alive (62.9% of neonates, 95% CI = 40.9%-82.4%, I^2^ = 94.6% and 69.6% of infants and young children, 95% CI = 59.6-78.7, I^2^ = 95.5%, **Figure 7**), though only a minority of those discharged were referred for further care (30.7% of neonates, 95% CI = 7.1%-61.3%, I^2^ = 94.7% and 34.9% of infants and young children, 95% CI = 15.1-57.9, I^2^ = 98.7%)). Most carers offered referral for their baby accepted it (65.9%, 95% CI = 49.3%-81.0%, I^2^ = 50.3%). The same was true for caregivers of infants and young children (69.3%, 95% CI = 57.7-79.9, I^2^ = 85.6%).

### Exploration of heterogeneity

Heterogeneity was high even within subgroups. This is common in meta-analyses of proportions. Meta-regression results are presented in **Table 3**. Heterogeneity in place of death was partly explained by user-fee policy (adjusted co-efficient for death at home 0.38 95% CI = 0.06-0.70, *P* = 0.020, R^2^ = 11.00%), whereas variance in care-seeking patterns was partly accounted for by age (adjusted co-efficient for whether care was sought outside the home 0.49, 95% CI = 0.21-0.77, *P* = 0.001) (see **Table 3** for each stage of the care pathway). Neither national income status nor user-fee policy was consistently associated with differences in care-seeking patterns (Tables S2 and S3 in **Online Supplementary Document**).

**Table 3:** Exploration of heterogeneity using meta-regression*

Outcome	Age group			Income status			User fees			Number of studies	Adjusted r2 (%)
	**Co-eff**	**95% CI**	***P*-value**	**Co-eff**	**95% CI**	***P*-value**	**Co-eff**	**95% CI**	***P*-value**		
Died at home	-0.02	-0.33, 0.30	0.918	0.06	-0.51, 0.39	0.780	0.38	0.06, 0.70	0.020	29	11.00
Died in a health facility	-0.13	-0.64, 0.38	0.601	0.25	-0.45, 0.94	0.466	-0.57	-1.09, -0.05	0.033	24	11.33
Died on route	Incalculable										
Died elsewhere	Incalculable										
Illness recognition	0.05	-0.04, 0.14	0.272	0.04	-0.05, 0.13	0.372	-0.04	-0.13, 0.05	0.342	17	11.51
Signs of severe illness	0.13	-0.20, 0.45	0.399	0.34	-0.15, 0.83	0.149	-0.16	-0.52, 0.19	0.322	13	1.13
Died immediately/no care given	-1.52	-2.48, -0.55	0.005	0.00	-1.52, 1.52	0.998	-0.10	-1.08, 0.88	0.833	17	37.29
Home care	1.18	0.43, 1.92	0.005	-0.20	-1.40, 1.00	0.726	0.18	-0.63, 1.00	0.632	16	39.87
Sought/attempted to seek care	0.53	0.27, 0.79	<0.001	-0.04	-0.57, 0.48	0.862	0.01	-0.25, 0.28	0.928	31	36.90
Sought/attempted to seek formal care	0.59	0.28, 0.90	0.001	0.03	-0.26, 0.31	0.851	-0.08	-0.39, 0.23	0.604	29	33.29
Sought/attempted to seek informal care	0.50	-0.30, 1.30	0.201	0.09	-0.54, 0.71	0.770	0.73	-0.08, 1.54	0.073	20	10.22
Died before setting out/on route to 1st provider	Incalculable										
Arrived at facility alive	0.47	-0.07, 1.01	0.082	0.24	-0.55, 1.03	0.499	-0.24	-0.84, 0.37	0.391	12	12.60
Left facility alive	0.67	0.05, 1.29	0.039	-0.10	-1.03, 0.82	0.785	0.46	-0.31, 1.24	0.184	9	53.15
Referred for further care	0.96	-0.09, 2.00	0.068	-0.89	-2.56, 0.78	0.257	0.33	-0.91, 1.56	0.565	13	22.01
Accepted referral	1.39	-0.13, 2.91	0.068	-1.33	-3.91, 1.24	0.266	1.05	-0.99, 3.10	0.269	12	21.28

CI – confidence interval

## DISCUSSION

### Summary of results

This review and meta-analysis demonstrated that approximately half of deaths of neonates, infants and young children occur at home in those countries for which place of death data are available across sub-Saharan Africa. In both age groups, signs and symptoms of illness were reported in the large majority of children (over 95%). However, whereas care-seeking for dying neonates is poor, many older children die at home despite their caregivers engaging formal care services before they die.

Forty percent of neonates received no care of any sort during their final illness. Only half of caregivers attempted to seek care outside the home, and fewer still sought formal health care. Only a third of all neonates arrived at formal health care facilities alive, though most studies which reported on this stage of the care pathway excluded neonates who were born and died in a health care facility without being discharged. It is probable that this would be much higher proportion if one were to include those who were alive *in utero* when the mother presented for delivery at the health facility. Health facilities discharged almost two thirds of the ill neonates, with less than a third of those who were discharged referred on for further care. These findings suggest that modifiable factors contributing to neonatal deaths include a failure of caregivers to recognise early signs of illness, or to appreciate the severity of neonatal illness (even if they report signs and symptoms of disease), which contribute to delays in seeking health care. Barriers to care-seeking resulting in even minor delays can be fatal as neonatal illness tends to progress rapidly which reduces the likelihood of reaching a facility alive compared to older children. However, high rates of discharge and low referral rates might suggest that when caregivers sought and received care it was inadequate.

In contrast, only a small minority of children older than one month died without receiving care of any sort. Some caregivers sought informal care, but almost three quarters of the children were taken to a formal health care provider and a majority arrived at that provider alive. Healthcare facilities discharged over two thirds of these children, and of those only a third were referred for further care. This would suggest that for older children, modifiable factors might be more concentrated at the health facility level.

Statistical heterogeneity was high, even across sub-groups. This is common in studies that pool proportions and continuous measures. Proportions are less stable between settings than ratio effect measures. Thresholds of I^2^ used for pooling trials are not appropriate for meta-analysis of continuous variables [48]. Furthermore, three factors may have contributed to the statistical heterogeneity across sub-groups: first, heterogeneity at certain stages of the care pathway may have arisen due to narrow confidence intervals for individual studies [48] eg, illness recognition by caregivers of infants and young children was high despite the total range of findings across all studies for post-neonatal deaths being 79.3%-100%. Similarly, the range reported for neonates being given home care was 5.1%-27.8%. The differences in these proportions are clinically small and would not change the practical interpretation of the results. Second, outlier studies contributed to heterogeneity at some stages of the care pathway; eg, for the proportion of infants and young children who died immediately or did not receive any care, the full range across 13 studies was 0.9%-19.9%, but there was one “outlier” study from Burkina Faso with a 95% confidence interval of 31.0% and 73.8% (**Figure 5**). Similarly, regarding the proportion of infants and young children whose parents sought/attempted to seek care, most studies ranged from 52.5%-98.9%, however the same study from Burkina Faso reported much lower rates of attempted care-seeking in the same age group (95% CI = 13.3%-53.1%). This was the smallest of all the studies we included (investigating only 17 deaths) and was also one of the earliest (conducted in 1999) in a very remote part of Burkina Faso, which probably explains the low levels of care-seeking and the very wide confidence interval. Third, while we tried to account for certain “macro” differences, such as national income and user-fee policy, these were nonetheless crude markers of known social determinants of health. We were unable to adjust for rural or urban setting as there was limited data from urban settings to use as a comparator (only 2 studies), and data on other known predictors of care-seeking behaviour - such as distance to clinics and levels of education - was simply unavailable consistently across the included studies. The question of context might explain the seemingly bimodal pattern of home deaths in settings with user-fees (Appendix S4 in in **Online Supplementary Document**): almost all deaths in the studies from Ethiopia [37], Kenya [18] and Guinea [20] appeared to happen at home. In Ethiopia, the authors suggested that the high proportions of home deaths might reflect high levels of unattended home births in this community, and limited access to health facilities due to terrain and location of villages far from main roads [37]. In Kenya, home deaths occurred despite a majority having sought care outside the home. Important contextual factors here were low follow-up and referral rates from formal health providers, underuse of community health workers and a preference for traditional healers [18]. In Guinea, home deaths were attributed to a combination of a lack of trained traditional birth attendants for home births, poor recognition of danger signs and poor care-seeking outside the home [20]. Both the studies in Kenya and Guinea were conducted in the late 1990s in rural areas, prior to the expansion of primary care initiatives such as the Integrated Management of Childhood Illness (IMCI); it is possible that care-seeking behaviour and place of death has changed in more recent years.

### Comparison with previous studies

Our findings are consistent with previous studies that highlighted failures to recognise signs of illness, particularly in neonates [49-51]. As our study included only children who died, care-seeking would have been appropriate for most if not all of them. Failure to provide or seek care for a child despite the presence of symptoms and signs of illness has been linked to low health literacy in the communities where these studies were conducted [35]. As noted by Koffi, Maina, Yaroh et al (2016), a lack of care-seeking could suggest that caregivers do not appreciate the significance of clinical features as markers of severe illness requiring urgent medical care [52]. Different conceptions of the aetiology of disease, and cultural beliefs regarding appropriate treatment providers also contribute to delays in care-seeking [43,53,54]. Low levels of maternal education may contribute to the overall mortality risk of children, and also to the specific care-seeking patterns demonstrated [4,45,52].

We also found relatively high rates of care-seeking during a child’s final illness – particularly for infants and young children. Multiple studies have commented on similar findings, emphasising that attention needs to be directed to modifiable factors at the health care facility level [5,17,23]. The low referral rates we found across both age groups have also been noted before, though without adequate explanation [4,52]. Some studies have suggested that barriers to referral include lack of transport, cost and distance to the referral facility [52], however our findings suggest these may not be insurmountable obstacles for caregivers as demonstrated by the high rates of referral acceptance when referral is made. Our findings are consistent with other studies which have suggested that staff who are the first point of contact, including community health workers and primary health care facility staff, might benefit from further training on the recognition of danger signs, and on familiarisation with indications for referral and the establishment of clear referral guidelines and pathways [4,18].

Because we have brought together all the available evidence, we were able to analyse factors across settings. No differences were noted in place of death or care-seeking behaviour between countries of different income status. User-fees were associated with a higher proportion of home deaths (69% in settings with user-fees as opposed to 44% in settings without user-fees), however this association is difficult to interpret given the bimodal distribution pattern for home deaths in countries with user-fees (as shown in Appendix S4 in **Online Supplementary Document**). Furthermore, no statistically significant differences were seen across the care pathway based on user-fees, suggesting that despite charges caregivers sought care - although there was a trend towards higher rates of seeking formal care in settings without user-fees (65%, compared to 53% in countries with user-fees), and higher rates of informal care in settings with user-fees (25%) compared to those without user-fees (11%). Many studies highlight cost as a barrier to care-seeking [35,52]. It is possible that user-fees also affect the decision to accept hospital admission, or caregivers’ abilities to follow home recommendations including the purchase of medications – neither of which we captured in this review, but which would warrant further investigation to understand how different health system financing models affect specific care-seeking decisions in childhood illness.

### Strengths and limitations

This study had three important strengths and corresponding limitations. First, we sought to pool studies from different settings in sub-Saharan Africa. This has contributed to the findings of high heterogeneity. Whilst this means it is unlikely that there would be a single numerical answer for every setting, it also allows us to draw conclusions with a broad evidence base and to compare the settings to attempt to understand reasons for the differences.

Second, we included only children who had died. This meant that there was no comparison made to children who survived, and so we cannot determine whether there are differences at specific stages of the pathway that might increase mortality risk. The strength of including only children who died is that it allows us to say that the included children should have had medical care sought and provided. It is likely that the children were seriously unwell so this should have been recognised, though it is possible that a small minority of children died from sudden infant death syndrome and so would have appeared well prior to death.

Finally, by pooling evidence across multiple study designs we were able to present data across each of the stages of the care pathways as defined by the Pathways to Survival framework. However, we were unable to disaggregate the databased on place of death, and so drawing direct conclusions about why high proportions of children die at home across sub-Saharan Africa remains difficult. In some studies, we were also unable to identify whether they included or excluded neonates who had arrived alive at a health facility *in utero*, and were delivered, but died prior to discharge.

### Implications and recommendations for policy

We found evidence consistent with neonates dying quickly or without recognition of their illness. The implication of these patterns is that health care systems should target all neonates for surveillance, irrespective of the concerns of parents. A system that provides monitoring of health for neonates could be a post-natal clinic or visitor scheme, or might be a target for technological solutions [55]. Health education interventions to improve caregiver recognitions of “danger signs” would also be of value. Unfortunately, it is possible that many of those children who reached formal care did not get adequate quality care, and many were discharged without referral. However, the Pathways to Survival framework is limited in its ability to assess care quality and the appropriateness of referral. Other sources of information including facility-based audits and confidential enquiries into child deaths are better placed to assess care quality and suggest strategies to improve care at the health facility level. High quality formal care might also improve the proportion of caregivers who seek care.

The majority of caregivers of older children sought and arrived at formal care, though informal and home care was inappropriately high as well. This is consistent with caregivers recognizing severe illness. We found a widespread pattern of discharging most of the children without referral, all of whom had an illness that ultimately killed them. This may be a function of poor quality care, which should serve as the focus for future interventions to address preventable mortality in infants and young children [56]. However, further work would also be required to fully understand whether the discharge of ill children is at the caregiver’s request which might reflect differences in traditional beliefs around preferred place of death or practical considerations such as the relative costs of transporting a terminally ill but alive child (which can be a normal taxi fare) compared to the much higher costs of transporting a dead body [57]. Caregivers may also want to avoid death occurring in a health facility in cases where the cause of death entered on the death certificate would cause stigma for the family, as has been noted in HIV/AIDS-related deaths [58,59].

While we found some evidence that user-fees may be associated with higher proportions of home deaths, the mechanism is unclear, and this effect appears to vary across settings. Other research has shown an increase in treatment-seeking for childhood illness from formal health care providers when user-fees are removed [60-62]. Further research is required to better understand the impact of user-fees in different contexts on care-seeking, referral and admissions, and subsequent place of death before policy recommendations can be made.

### Implications for clinical practice

Clinicians working in low-and-middle income settings across sub-Saharan Africa should be aware that a large proportion of the children who die are seen by clinical staff and sent home. They should therefore consider clear safety-netting advice for caregivers, with instructions for what should prompt re-presentation.

### Implications for research

Researchers conducting population-based research should consider reporting deaths according to the Pathways to Survival framework, and so should ensure that the data collection tools used to investigate deaths include the necessary information to report on each stage of the pathway. It is also important to standardise definitions for each stage of the care pathway and the questions used to calculate the relevant numerator and denominator. For example, illness recognition may refer to the presence or reporting of symptoms in response to a verbal-autopsy questionnaire or it may require that caregivers indicate that such symptoms were indicative of illness requiring medical attention. Similarly, when asking whether care was sought outside the home, it is necessary to clarify whether this includes caregivers who intended to seek care but were delayed or unable to reach a provider. With regards to neonatal deaths, reporting should be standardised to either include or exclude neonates who were born and died in a health facility without being discharged. The definitions used in this study (available as Appendix S5 in **Online Supplementary Document**) might serve as a starting point for those undertaking new research.

Finally, studies should be designed to capture representative samples and take action if there are reasons to suspect this has not been done. Our modified Axis tool could be used for assessment of study design for the design, reporting and assessment of future studies of under-5 deaths. Further studies of under-5 deaths should be carried out in under-represented areas of sub-Saharan Africa (**Figure 2**) and should include data on quality of care in addition to care-seeking behaviour.

## CONCLUSIONS

This study is the first to present a common picture for care-seeking behaviour in fatal childhood illness across sub-Saharan Africa, across each of the stages of the Pathways to Survival framework. Despite high heterogeneity, findings nevertheless suggest that there are differences in care-seeking behaviour between neonatal deaths and deaths of infants and young children. Understanding such care-seeking patterns is important in highlighting targets for interventions to reduce under-5 mortality across the region, including the need to improve recognition of danger signs in neonates, increasing the quality of care for both neonates and children 1-59 months and ensuring safety-netting, appropriate referral and improved post-discharge counselling and follow-up by health care providers.

## Additional material

Online Supplementary Document
