# Supplementary material for: Place of death, care-seeking and care pathway progression in the final illnesses of children under five years of age in sub-Saharan Africa: a systematic review
Source: J Glob Health. 2019 Oct 22;9(2):020422. doi: 10.7189/jogh.09.020422 (PMC6815655; doi:10.7189/jogh.09.020422)
Supplement: Online Supplementary Document [file jogh-09-020422-s001.zip › Appendix 2 - summary of results by national income status_JOGH.docx]

| **Table S2: summary of results, stratified by national income status** | | | | | | | | | | | |
| --- | --- | --- | --- | --- | --- | --- | --- | --- | --- | --- | --- |
| **Outcome** | **Low income** | | | | **Lower middle income** | | | | **Upper middle income** | | |
|  | Number of studies* | Number of deaths | Proportion  (95% CI) | I^2^ | Number of studies* | Number of deaths | Proportion  (95% CI) | I^2^ | Number of studies* | Number of deaths | Proportion (95% CI) |
| Died at home | 22 | 5980 | 53.3 (44.0-62.5) | 98.0 | 4 | 3196 | 55.4 (37.9-72.1) | 98.6 | 0 | - | - |
| Died in a health facility | 17 | 5330 | 35.2 (26.4-44.6) | 97.9 | 4 | 3196 | 34.9 (20.8-50.4) | 98.3 | 0 | - | - |
| Died *en route* to a health facility | 7 | 1718 | 5.7 (2.6-9.9) | 88.2 | 3 | 2856 | 3.5 (2.6-4.6) | - | 0 | - | - |
| Died elsewhere | 12 | 3562 | 4.7 (1.7-9.1) | 96.0 | 4 | 3196 | 5.8 (1.8-11.6) | 96.1 | 0 | - | - |
| Signs/Symptoms of illness | 10 | 3736 | 98.0 (93.6-100.0) | 97.7 | 3 | 2793 | 100.0 (100.0-100.0) | - | 1 | 110 | 95.5 (89.8-98.0) |
| Signs/Symptoms of severe illness | 9 | 3307 | 87.4 (74.8-96.2) | 98.8 | 3 | 2793 | 97.0 (96.3-97.6) | - | 0 | - | - |
| Died immediately/no care given | 15 | 4033 | 15.2 (7.7-24.4) | 98.1 | 3 | 2793 | 18.2 (4.9-37.3) | - | 0 | - | - |
| Home care given | 12 | 3969 | 44.9 (27.5-63.0) | 99.1 | 3 | 2793 | 28.9 (16.1-43.7) | - | 0 | - | - |
| Sought/attempted to seek care outside the home | 27 | 6949 | 71.2 (62.7-79.1) | 98.2 | 3 | 2793 | 73.3 (54.3-88.6) | - | 0 | - | - |
| Sought/attempted to seek formal care | 23 | 6136 | 61.1 (51.0-70.7) | 98.3 | 3 | 2793 | 61.2 (39.4-80.8) | - | 1 | 110 | 61.8 (52.5-70.4) |
| Sought/attempted to seek informal care | 13 | 4610 | 16.2 (8.1-26.4) | 98.6 | 3 | 2793 | 22.1 (9.6-38.0) | - | 1 | 110 | 17.3 (11.3-25.4) |
| Died before setting out/*en route* to provider | 8 | 2527 | 8.8 (4.8-13.7) | 91.7 | 3 | 2793 | 9.6 (2.6-20.3) | - | 0 | - | - |
| Arrived alive at formal health facility (out of all deaths) | 9 | 2857 | 50.3 (38.9-61.7) | 97.0 | 3 | 2793 | 50.0 (24.9-75.2) | - | 0 | - | - |
| Arrived alive at formal health facility (of those who sought formal care) | 9 | 1679 | 87.9 (79.3-94.6) | 94.1 | 3 | 1852 | 83.4 (63.7-96.5) | - | 0 | - | - |
| Left formal health facility alive (out of all deaths) | 6 | 2484 | 33.0 (21.0-46.1) | 97.7 | 3 | 2793 | 35.5 (14.5-60.1) | - | 0 | - | - |
| Left formal health facility alive (of those who arrived alive) | 6 | 1245 | 67.7 (51.5-82.0) | 96.9 | 3 | 1406 | 71.4 (58.6-82.6) | - | 0 | - | - |
| Referred for further care (out of all deaths) | 9 | 2985 | 16.7 (8.9-26.1) | 97.3 | 3 | 2793 | 5.8 (1.5-12.3) | - | 0 | - | - |
| Referred for further care (of those who left the facility alive) | 4 | 721 | 31.0 (6.5-63.3) | 98.5 | 3 | 967 | 15.1 (9.8-21.2) | - | 0 | - | - |
| Accepted referral (of all deaths) | 8 | 2888 | 10.6 (5.3-17.4) | 96.1 | 3 | 2793 | 4.4 (1.1-9.7) | - | 0 | - | - |
| Accepted referral (of those referred) | 8 | 521 | 63.0 (51.9-73.6) | 77.3 | 3 | 140 | 83.2 (75.9-89.6) | - | 0 | - | - |

*Note: In cases where data was collected from more than one country as part of the same study, each country was counted as a separate study for the purpose of the meta-analysis

# Only 1 study therefore I^2^ not reported.

Figures in red: significant at P<0.05; figures in orange: significant at p<0.1

| **Table S3: summary of results, stratified by whether or not user fees are charged for health services** | | | | | | | | |
| --- | --- | --- | --- | --- | --- | --- | --- | --- |
| **Outcome** | **User fees** | | | | **No user fees** | | | |
|  | Number of studies* | Number of deaths | Proportion  (95% CI) | I^2^ | Number of studies* | Number of deaths | Proportion  (95% CI) | I^2^ |
| Died at home | 9 | 3630 | 69.1 (56.2-80.6) | 98.4 | 16 | 3364 | 43.8 (34.3-53.5) | 96.7 |
| Died in a health facility | 8 | 3533 | 24.9 (14.6-36.9) | 98.3 | 11 | 2811 | 44.8 (33.5-56.5) | 97.3 |
| Died *en route* to a health facility | 2 | 799 | 3.6 (2.4-5.0) | - | 6 | 1593 | 5.2 (1.9-9.7) | 89.6 |
| Died elsewhere | 6 | 2470 | 5.6 (0.8-13.9) | 97.9 | 8 | 2106 | 3.9 (1.9-6.5) | 84.3 |
| Signs/Symptoms of illness | 5 | 2250 | 96.9 (89.6-100.0) | 97.8 | 8 | 2333 | 99.1 (95.1-100.0) | 96.9 |
| Signs/Symptoms of severe illness | 5 | 1962 | 89.6 (70.9-99.4) | 99.0 | 6 | 2082 | 89.6 (76.3-97.9) | 98.4 |
| Died immediately/no care given | 8 | 2120 | 19.4 (9.1-32.4) | 97.4 | 8 | 2536 | 15.2 (4.9-29.7) | 98.7 |
| Home care given | 5 | 1846 | 38.9 (27.0-51.5) | 95.7 | 9 | 2587 | 44.9 (22.6-68.2) | 99.3 |
| Sought/attempted to seek care outside the home | 11 | 3942 | 67.9 (52.5-81.5) | 98.9 | 17 | 3630 | 70.9 (60.7-80.2) | 97.5 |
| Sought/attempted to seek formal care | 12 | 3945 | 53.1 (39.0-66.9) | 98.6 | 13 | 2915 | 65.0 (51.6-77.3) | 98.0 |
| Sought/attempted to seek informal care | 6 | 3037 | 25.2 (14.4-37.8) | 98.2 | 10 | 2420 | 11.3 (4.2-21.0) | 97.5 |
| Died before setting out/*en route* to provider | 4 | 1516 | 7.6 (3.8-12.6) | 84.0 | 5 | 1634 | 8.0 (2.8-15.5) | 94.7 |
| Arrived alive at formal health facility (out of all deaths) | 5 | 1846 | 45.4 (27.9-63.5) | 98.0 | 5 | 1634 | 49.6 (32.0-67.3) | 97.9 |
| Arrived alive at formal health facility (of those who sought formal care) | 5 | 1185 | 92.1 (80.5-99.2) | 95.2 | 5 | 940 | 84.8 (73.3-93.6) | 93.3 |
| Left formal health facility alive (out of all deaths) | 3 | 1499 | 40.0 (22.4-59.0) | - | 4 | 1608 | 26.7 (11.7-45.0) | 98.3 |
| Left formal health facility alive (of those who arrived alive) | 3 | 898 | 78.2 (75.4-80.9) | - | 4 | 758 | 65.4 (39.0-87.6) | 97.9 |
| Referred for further care (out of all deaths) | 4 | 1596 | 12.3 (4.1-23.8) | 96.7 | 7 | 2126 | 16.3 (7.4-27.6) | 97.3 |
| Referred for further care (of those who left the facility alive) | 3 | 700 | 34.4 (4.5-73.9) | - | 4 | 498 | 34.5 (7.6-68.4) | 97.8 |
| Accepted referral (of all deaths) | 3 | 1499 | 9.1 (3.0-17.9) | - | 7 | 2126 | 9.5 (4.3-16.4) | 94.9 |
| Accepted referral (of those referred) | 3 | 278 | 73.5 (58.0-86.7) | - | 7 | 315 | 62.3 (48.0-75.7) | 77.6 |

*Note: In cases where data was collected from more than one country as part of the same study, each country was counted as a separate study for the purpose of the meta-analysis

Figures in red: significant at P<0.05; figures in orange: significant at p<0.1
