# Supplementary material for: Place of death, care-seeking and care pathway progression in the final illnesses of children under five years of age in sub-Saharan Africa: a systematic review
Source: J Glob Health. 2019 Oct 22;9(2):020422. doi: 10.7189/jogh.09.020422 (PMC6815655; doi:10.7189/jogh.09.020422)

Appendix 4: Proportion of children who died at home by national income and user fee policy

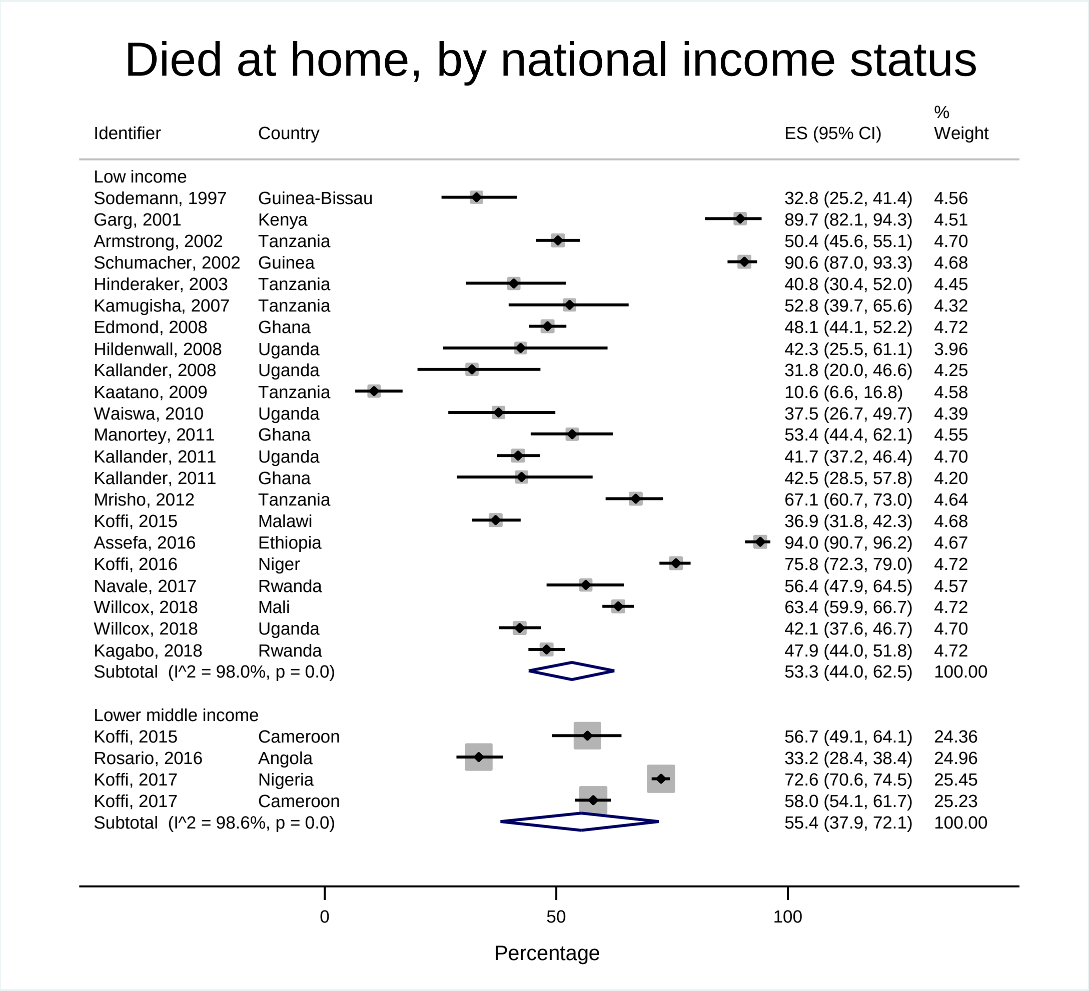

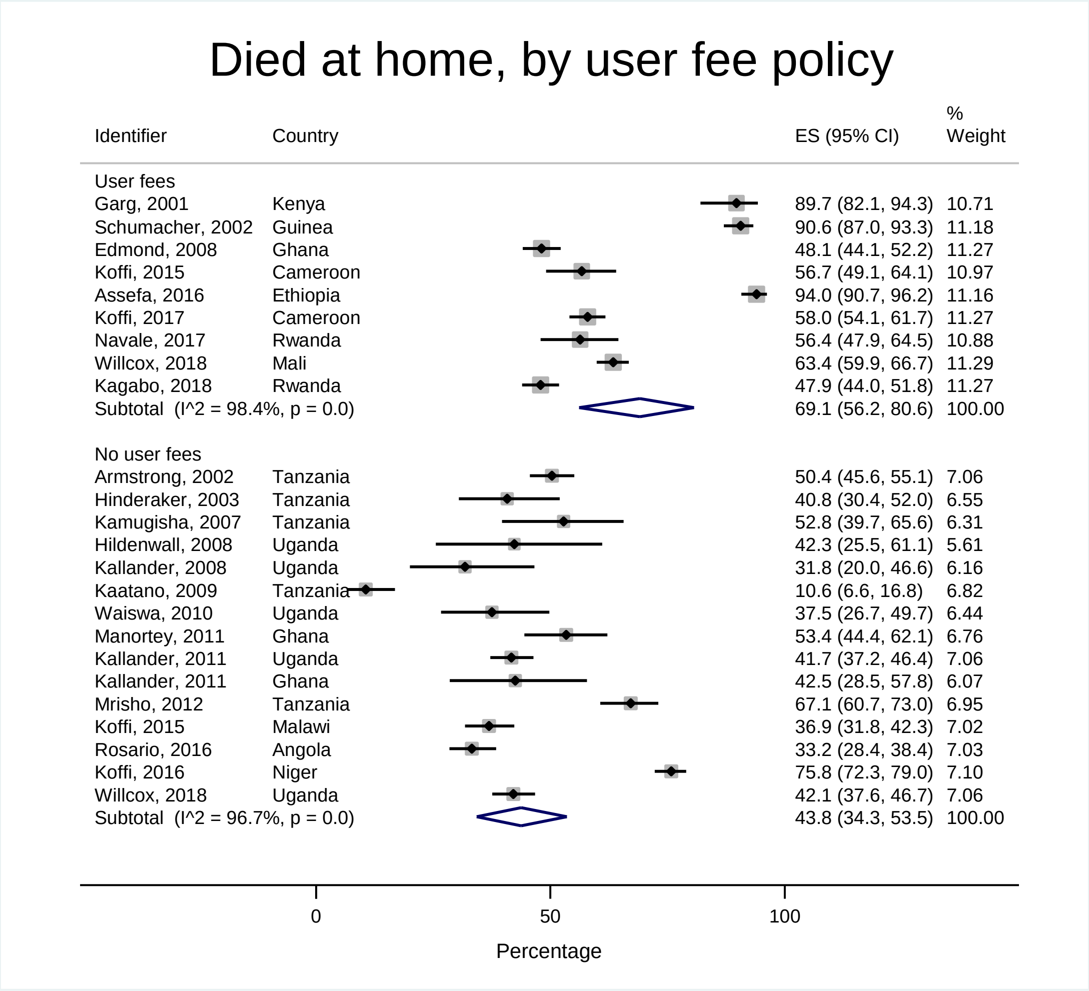

Supplement: Online Supplementary Document [file jogh-09-020422-s001.zip › Appendix 4.pdf]
