# Supplementary material for: Place of death, care-seeking and care pathway progression in the final illnesses of children under five years of age in sub-Saharan Africa: a systematic review
Source: J Glob Health. 2019 Oct 22;9(2):020422. doi: 10.7189/jogh.09.020422 (PMC6815655; doi:10.7189/jogh.09.020422)
Supplement: Online Supplementary Document [file jogh-09-020422-s001.zip › Appendix 5 - suggested definitions for each stage of the care pathway.docx]

| **Appendix 5: Suggested definitions for each stage of the care pathway** | |
| --- | --- |
| **Term/Stage of care pathway** | **Definition** |
| Died at home: | # who died at home / total deaths |
| Died in any formal health facility: | # who died in a health facility (primary, secondary or tertiary level) / total deaths |
| Died en route to a health facility | # who died en route to a health facility (primary, secondary or tertiary level) / total deaths |
| Died elsewhere/other place of death | Place of death not at home, in a health facility or en route to a health facility / total deaths |
| Illness recognition | # Any signs or symptoms of illness reported/total deaths |
| Severe illness recognised | # of deceased children whose caregivers reported one or  more possibly severe or severe signs or symptoms/total death  Severe illness was defined by  the caretakers’ mention of at least one of the danger  signs defined by the Integrated Management of Childhood  Illness (IMCI) guidelines. These include convulsions,  chest indrawing, nasal flaring, grunting,  bulging fontanelle, umbilical redness extending to the  skin, many or severe skin pustules, lethargic or unconscious  or less than normal movement, not able to drink  or breastfeed, vomits everything, convulsions, loose  stools/diarrhea > 2 days, heavy bleeding |
| No care given/did not attempt to seek care. | # with no home care and no attempt to seek care outside the home / total deaths |
| Home care given | # Home care given / total deaths  (Note: no judgment made of appropriateness of home care) |
| Care sought/attempted to seek care outside the home | Sought or attempted to see any  Care outside the home for the child’s fatal illnesses / total death  (Note this includes formal and informal sources of care) |
| Sought/attempted to seek formal care | Sought or attempted to seek formal care / total deaths |
| Died before setting out for formal care/en route/could not reach ANY formal provider | Died before setting out or en route to formal provider / total deaths |
| Sought/attempted to seek Informal care | Sought or attempted to seek informal care / total deaths |
| Proportion who leave the first health facility alive: | # of children leaving the health facility alive / total deaths |
| Proportion who leave the first health facility alive (of those who arrived alive): | # of children leaving the health facility alive / number who arrived at the facility alive |
| Referral from first facility | # of children referred for further care / total deaths |
| Referral from first facility (of those discharged from the facility alive) | # of children referred for further care / number of those who leave the facility alive |
| Acceptance of referral | # of caregivers who accept the referral of their child / total deaths  (Note: this includes children who died before reaching the referral facility) |
| Acceptance of referral (of those who were referred for further care) | # of caregivers who accept the referral of their child / number referred for further care |
